# The Protective Effect of Anthocyanins Extracted from *Aronia Melanocarpa* Berry in Renal Ischemia-Reperfusion Injury in Mice

**DOI:** 10.1155/2021/7372893

**Published:** 2021-01-22

**Authors:** Li Li, Jun Li, Hui Xu, Fengmei Zhu, Zhijun Li, Hongzhi Lu, Jinrong Zhang, Zhengsheng Yang, Yongsheng Liu

**Affiliations:** ^1^Department of Internal Medicine, The First Hospital of Qinhuangdao, No. 258 Wenhua Road, Haigang, Qinhuangdao, Hebei, China 066000; ^2^Hebei Normal University of Science and Technology, No. 360, Hebeidajie, Haigang, Qinhuangdao, Hebei, China 066000; ^3^The People Hospital of Shanhaiguan, No. 5, Guancheng Road, Shanhaiguan, Qinhuangdao, Hebei, China 066200

## Abstract

**Background:**

Our previous research showed the antioxidant activity of anthocyanins extracted from *Aronia melanocarpa* of black chokeberry in *vitro.* Ischemia acute kidney injury is a significant risk in developing progressive and deterioration of renal function leading to clinic chronic kidney disease. There were many attempts to protect the kidney against this progression of renal damage. Current study was designed to examine the effect of pretreatment with three anthocyanins named cyanidin-3-arabinoside, cyanidin-3-glucodise, and cyaniding-3-galactoside against acute ischemia-reperfusion injury in mouse kidney.

**Methods:**

Acute renal injury model was initiated by 30 min clamping bilateral renal pedicle and followed by 24-hour reperfusion in C57Bl/6J mice. Four groups of mice were orally pretreated in 50 mg/g/12 h for two weeks with cyanidin-3-arabinoside, cyanidin-3-glucodise, and cyaniding-3-galactoside and anthocyanins (three-cyanidin mixture), respectively, sham-control group and the renal injury-untreated groups only with saline.

**Results:**

The model resulted in renal dysfunction with high serum creatinine, blood urea nitrogen, and changes in proinflammatory cytokines (TNF-ɑ, IL-1*β*, IL-6, and MCP-1), renal oxidative stress (SOD, GSH, and CAT), lipid peroxidation (TBARS and MDA), and apoptosis (caspase-9). Pretreatment of two weeks resulted in different extent amelioration of renal dysfunction and tubular damage and suppression of proinflammatory cytokines, oxidative stress, lipid peroxidation, and apoptosis, thus suggesting that cyanidins are potentially effective in acute renal ischemia by the decrease of inflammation, oxidative stress, and lipid peroxidation, as well as apoptosis.

**Conclusion:**

the current study provided the first attempt to investigate the role of anthocyanins purified from *Aronia melanocarpa* berry in amelioration of acute renal failure via antioxidant and cytoprotective effects.

## 1. Introduction

*Aronia melanocarpa* is a native deciduous shrub in the northeastern United States. Several lines of evidence support a potential therapeutic role for the extracts of black chokeberry in regulation of platelet adhesion and aggregation [1], pancreatic ɑ-amylase and lipase [2], and chemopreventive benefit [3]. With the improvement of purification technology, the extracts of black chokeberry have been well recognized as optional natural resources in health care [4, 5].

Evidence showed that anthocyanins have a powerful antioxidant activity among natural pigments from berry extracts [6–9], suggesting that chokeberry fruits can be considered as promising food candidate with enhanced antioxidant potential. As a natural resource both of medicine and food, the chokeberry fruit also contains extremely rich anthocyanins, one of members of the flavonoid category of phenolics. In the investigation of Concord and Salvador grape fruits which are the important source of grapes, Wang et al. reported several cyanidins including cyanidin-3-arabinoside and cyanidin-3-glucoside isolated from anthocyanins [10]. Jakobek et al. indicated a positive correlation between the antioxidant activity and total anthocyanin content in chokeberry fruit juice [11]. Furthermore, Rop et al. [12] reported that a high correlation between polyphenolic substances and antioxidant capacity, radical oxygen species scavenging, and lipid peroxidation inhibits activities in vitro. These substances included cyanidin-3-arabinoside, cyanidin-3-galactoside, (-) epicatechin, chlorogenic, and neochlorogenic acids. Recent study indicated that 8-week oral intake of extracts of *Aronia melanocarpa* increased antioxidant activity within liver and kidney in vivo [13].

While these previous studies suggested that the chokeberry fruit is one of main resource of anthocyanins and a significant antioxidant capacity of anthocyanins, data related to the effect of individual anthocyanin is largely lacking. By using UV-Vis, HPLC-DAD, and UPLC-MS technologies, we analyzed the components of *Aronia melanocarpa* anthocyanins and further identified four main constituent parts of the anthocyanins, including cyanidin-3-arabinoside, cyanidin-3-glucoside, cyaniding-3-galactoside, and cyaniding-3-xyloside (Figure 1 in Supplemental material) [14]. Our preliminary experiments in vitro suggested cyanidin-3-arabinoside acted as the most active component in both of the antioxidant ability and free radical scavenging activity (data not shown).

Acute kidney injury (AKI) is a common clinical manifestation defined by an abrupt (<48 h) increase in serum creatinine resulting from an injury or impact leading a functional decline or structural change in the kidney [15]. Renal ischemia/reperfusion injury is defined as a generalized or localized impairment of oxygen and nutrient supply and waste product removal from the kidney [16]. It is a common cause of AKI and presents approximately 80–90% of the renal etiologies and is associated with worse clinical outcomes, including end of kidney failure and high mortality and postoperative care costs [17].

From the epidemiological perspective, there is an association between broad syndromes and various etiologies [18]. While the course of renal ischemia/reperfusion injury is not completely understood, data suggest that the injury augments renal function deterioration by multiple potential mechanisms, such as endothelial dysfunction, increased oxidative stress, and infiltrating inflammation as well as stimulating prominent cytokines [19, 20].

In myocardial ischemia reperfusion injury, reperfusion further aggravates the ischemic injury by interrupting the respiratory chain, especially mitochondria function [21], suggesting that preconditioning prior to ischemia or reperfusion confers protections of the physiological and biochemical functions of mitochondria [22]. In ischemia reperfusion injury of the isolated kidney, simultaneous addition of the oxygen metabolite scavenger-catalase may prevent the decline of GFR [23]. However, pathological consequences from reperfusion injury may initiate within seconds of reoxygenation; it is not easy to manage and cure the renal dysfunction. Therefore, early prevention and management of the patients at high risk of acute kidney injury are paramount [24]. There are increasing evidences of clinical strategy of acute kidney injury prevention as a practice guideline recommendation [25, 26].

Although some *in vitro* models provide evidence for the features of renal ischemia and reperfusion pathology, they cannot provide the sufficient insight into precise environment *in vivo* [4]. Therefore, we designed this *in vivo* study to investigate the potential role of three of identified anthocyanins including cyanidin-3-arabinoside, cyaniding-3-galactoside, and cyanidin-3-glucoside against renal ischemia-reperfusion injury in mice. The hypothesis of the current study is that anthocyanins inhibit oxidative stress, the signaling of multiple cytokines, and lipid peroxidation occurrence.

The investigation was particularly important because the previous observations were based on normal cells or tissues models, with no intervention for acute ischemia and reperfusion *in vivo*.

The findings from the present work not only indicated a nephroprotective role of cyanidins against ischemia-induced nephropathy, but also suggested anti-inflammatory, antioxidant, and antilipid peroxidation properties are ascribed to anthocyanin protection.

## 2. Materials and Methods

### 2.1. Black Chokeberry Collection and Anthocyanin Extraction

Black chokeberry fruits were harvested in full ripeness, of which 35 were randomly chosen from each of ten plants for extraction and analyses. The extraction and analyses were processed immediately after the harvest. Extraction was performed according to the microwave-assisted extraction methods [27–29] with some modification, using the following procedure: 140 g of a fresh fruit sample was homogenized by an electrical blender for 25 seconds, and then the resulting paste was put into a conical flask which contained with 250 ml 50% of ethanol. Next, the mixture was extracted in a microwave extraction reaction workstation (Model MAS-II, SINEO Microwave Chemistry Technology Co. Ltd., Shanghai, China) equipped with a timer and a temperature controller as well as a cooling system. The operating frequency was 2450 MHz with 470 W of a designed extraction power, and the extraction time was 8 min. The final solution in the flask was centrifuged at 4750 g × 10 min, and the collected supernatant was then filtered through a 0.45 *μ*m syringe filter and stored at 4°C before loading into the HPLC-DAD system.

### 2.2. Isolation and Identification of Anthocyanin Compounds from Anthocyanins

One hundred milliliters of aqueous anthocyanin extract was passed through an HP-20 resin column (Macroporous Adsorption Resin-M0043, Solarbio Life Sciences, Beijing, China). The optimal conditions for the isolation were determined as follows: elution solvent by 40% ethanol (contains 0.5% acetic acid) and pH 2.0, the adsorption conditions under 0.6 mg/ml, and the adsorption and desorption flow rates at 1 ml/min. The different cyaniding compounds were separated through an Eclipse XDB-C18 (4.6 × 150 mm and 5 *μ*m) chromatographic column within an Agilent 1260 Infinity Quaternary LC (Agilent, Santa Clara, US). The mobile phase was composed of solvent A (2.5% acetic acid aqueous solution) and solvent B (water/methanol/acetonitrile/acetic acid as 40/25/32.5/2.5, v/v/v/v). The main gradient elution conditions were as follows: 0–10 min, 5-8% solvent B (starting with 5% solvent B and ending with 8% solvent B); 10–20 min, linear gradient 8-12% solvent B; 20–35 min, linear gradient 12-90% solvent B; and 3-50 min, linear gradient 90% solvent B. Other parameters included flow rate (1 ml/min), column temperature (30°C), sample injected (5.0 *μ*l), and detection wavelength (520 nm). The mass spectrometric analysis was performed for identification of cyanidin compounds using a LTQ Orbitrap mass spectrometer (Thermo Fisher Scientific, Grand Island, NY, US). The mass spectral conditions were briefly shown as follows: ESI, positive ion mode; capillary voltage, 3.5 kV, desolvation temperature, 320°C; and desolvation pressure, 17 psig. Continuous mass spectra recoding was arranged from 100 to 1000 m/z with a scan time of 1 min and an interscan delay of 0.20 sec. Selected ion monitoring (SIM) mode was performed in the mass spectral acquisition. [M+H]^+^ ions of cyanidin-3-arabinoside and cyaniding-3-xyloside were observed at m/z 419, and cyaniding-3-galactoside and cyanidin-3-glucoside were indicated at m/z 449.

### 2.3. Animals and Oral Administration of Anthocyanins

All animals are handled, and procedures are performed in adherence to the Chinese National Health guide for the Care and Ethics of Laboratory Animals [30, 31], and all protocols are approved by Qinhuangdao first hospital research committee.

Male C57BL/6 mice 10–12 weeks old were obtained from the National Breeder Center of Rodents (Shanghai, China). They were housed in the facility under specific pathogen-free conditions, were allowed free access to standard chow (contained 13.45% protein, 51.6% carbohydrate, 3.40% fat, and 2908 kcal/kg of metabolizable energy) and water, and were kept in a 12 h light : 12 h dark cycle. All mice were acclimated to this environment for at least 1 week prior to the experiment. In the first set of experiments, 26 mice were randomly divided into two groups: the first one is sham-control (control) and the other undergoes 30 min of bilateral ischemia followed by reperfusion for 24 hours (IR). For the second set of experiments, 52 animals were randomly divided into four groups treated by chokeberry anthocyanin compounds, i.e., anthocyanin mixture (AC) cyanidin-3-arabinoside (C-3-A), cyaniding-3-galactoside (C-3-GA), and cyanidin-3-glucodise (C-3-GL), respectively (*n* = 13 each subgroup).

All anthocyanin compounds including cyanidin-3-arabinoside, cyanidin-3-glucodise, and cyaniding-3-galactoside were well solved in sterile 0.9% saline by 2 : 1 (mg/ml) with the final concentration of 0.5 mg/ml in each cyaniding. The anthocyanin mixture was a mixture of three compounds with ratio of 1 : 1 : 1 to make a final concentration of 0.5 mg/ml. All the solutions were kept at 4°C in dark bottles.

The mean of the administration was a polyurethane feeding tube with a rounded tip (16 ga × 38 mm, Instech Laboratories, Inc, Plymouth Meeting, PA, USA). Treated mice would be orally pretreated with the different anthocyanins in the dose of 50 mg/g body weight corresponding to 25 ml/g body weight in saline twice daily for 14 days prior to renal IR injury. Sham-control group and the renal IR-untreated (control) group in the first set were received equal volume of saline, i.e., 25 ml/g body weight before IR. We did not observe signs of regurgitation or bleeding after feeding. There was no abnormal behavior and symptom observed for the periods indicated.

### 2.4. Body Weight Measurement

Body weights of all animals were measured at 8 : 30 before feeding by a weighing scale of electronic digital balance with accuracy of 0.1 gram (Pushton, Henan, China). The recording times included the day (24 hours) prior to administration of anthocyanins, the day before renal IR injury operation, and immediately (within 5 minutes) after the operation.

### 2.5. Renal IR Injury Induction

We adapted an established mouse model of “warm” renal IR injury as previously described [32]. Briefly, mice were anesthetized with ketamine/xylazine (90/10 mg/kg or to effect, i.p.) and kept on a homoeothermic station to maintain body temperature at 37°C. A warming pad was used throughout the procedure until mice were awake, and 0.5-1.0 ml of sterile 0.9% saline by intraperitoneal injection was administrated to ensure the body temperature under control and fluid balance. A midline incision was made, and bilateral renal pedicles were exposed. Renal IR injury was induced by clamping both renal pedicles with atraumatic micro straight clamps. After 30 min of warm ischemia, clamps were removed to initiate renal reperfusion. Sham-control animals were subjected to identical operation except the clamp was not applied. The abdomen was closed. Mice were sacrificed at 24 h after reperfusion for blood and kidney tissue samplings under isoflurane-induced terminal anesthesia. Blood sample was taken by intracardiac puncture after accessing the chest cavity from underneath the diaphragm. Eighty-five to 95 *μ*l of serum was isolated and stored at −80°C until analysis was performed. The kidneys were harvested after euthanasia/cardiac puncture and cut in half along the renal pelvis. One kidney was immediately snap-frozen in liquid nitrogen while the other is fixed immediately. To avoid intertissue variability for histopathology evaluation, the same anatomical part for all mice was divided up to compare the same region of the fixed kidney.

### 2.6. Serum Analysis for Renal Functions

Serum creatinine (sCr) and blood urea nitrogen (BUN) levels were measured by the use of QuantiChromTM assay kits (DICT-500 for sCr and DIUR-100 for BUN) from Bioassay Systems (BioAssay Systems, Hayward, CA) following the manufacturer's protocol and previously described [33, 34]. First, all the blood samples were taken by intracardiac puncture under anesthesia as described in Section 2.5. Then, the samples were left in room temperature for 30 minutes. Finally, the serum was obtained after centrifugation of 1500 x g for 10 minutes at 4°C. The serum was transferred into a sterile empty tube and kept at 4°C for the subsequent assay. All the serum samples were measured in duplicate with a clear bottom 96-well plate. The sCr and BUN concentrations in the blood sample were directly proportional to the intensity of the color read at 510 nm and 520 nm, respectively. The concentrations were expressed as micromole per liter for serum creatinine and milligrams per deciliter for BUN.

### 2.7. Proinflammatory Cytokine Level Measurement

The serum levels of proinflammatory cytokines including tumor necrosis factor-*α* (TNF-*α*), interferon 1-*β* (IL-1*β*), interleukin-6 (IL-6), and monocyte chemoattractant protein-1 (MCP-1) were measured by Proteome Profiler Mouse Cytokine Array Kit, Panel A (R&D Systems, catalog number ARY006, Minneapolis, MN). The method was described previously [35], and the serum for cytokine array was obtained from the blood as described in Section 2.6. Fifteen microliters of serum from each animal was in duplicate for quantitative analysis of these cytokines according to the manufacturer's protocol. The kidney levels of these cytokines were determined by enzyme-linked immunosorbent assay (ELISA) kits (Biotrak ELISA System, Amersham Biosciences, NJ, USA, for IL-1*β* and TNF-*α* and Wuhan Elabscience Biotechnology Co., Ltd., China, for IL-6I and MCP-1) according to the manufacturer's protocols. The method was described previously with some modifications [36, 37]. Briefly, the kidney sample from the different study groups was cut into pieces and gently homogenized in 1/5 (w/v) 50 mmol/L (pH 7.4) ice-cold phosphate-buffered saline solution (PBS) containing a protease inhibitor cocktail (Sigma-Aldrich, St. Louis, MO) with 10 strokes at 1250 rev/min. Then, the supernatant for the assay was obtained after centrifugation at 4000 rpm for 10 min.

All procedures were repeated three times to guarantee the accuracy of the results according to the manufacturer's instructions. Protein contents were measured by using the BCA assay kit (Nanjing Jiancheng Biotechnology Co., Ltd). The results were expressed as picograms per milliliter (pg/ml).

### 2.8. Renal Proinflammatory Cytokines and Toll-Like Receptor 4 (TLR4) mRNA Expression

The kidney samples anatomically containing the same amount of cortex and medulla from each mouse were used for total RNA extraction through a rotor-stator homogenizer and treated with TRIzol reagent (Life Technologies, Carlsbad, CA) according to the manufacturer's protocols. A noncolumn DNAse I digestion (Life Technologies, Carlsbad, CA) was subsequently performed to remove genomic DNA in the extraction according to the manufacturer's instructions. Reverse transcription and real-time polymerase chain reaction (PCR) were processed through about 1.0 *μ*g of DNA-free RNA, with gene-specific primers and probe through iTaq™ Universal SYBR® Green One-Step Kit (Bio-Rad Lab, Hercules, CA) in ABI Prism 7700 Sequence Detection System (Applied Biosystems, Foster City, CA). The main thermal cycling conditions were 50°C for 2 minutes and 95°C for 10 minutes, followed by 45 cycles of 95°C for 15 seconds and 60°C for 1 minute.

The primer sequences of the analyzed genes including IL-1*β*, IL-6, TNF-ɑ, and MCP-1 as well as TLR4 were designed by Applied Biosystems' Primer Express Software, and its sequences are shown in Table 1.

The data for transcript levels of genes were subsequently quantitated in RT-PCR under fluorescence detection of SYBR green according to the manufacturer's instructions. All mRNA levels were normalized against GAPDH mRNA level and endogenous control and calculated with the comparative *Ct* method [38]. Each gene was run in triplicate in real-time PCR experiments.

### 2.9. Measurement of Renal Antioxidant Parameters

To evaluate renal antioxidant defense system, the activities of two antioxidant compounds–superoxide dismutase (SOD) and catalase (CAT)—were determined. First, the kidney sample was processed by homogenization as described in Section 2.7. Identified supernatant aliquots were subsequently used to determine SOD and CAT activities according to the method as described previously [39, 40]. The details of each test were the following in brief. (1) 50 *μ*ml of supernatant was mixed with 2.9 ml of solution A of 50 mmol/L PBS (pH 7.4) (containing 0.1 mmol/L EDTA and 2 mmol/L cytochrome c and 5 mmol/L xanthine) and then reacted with 50 ml of solution B (containing 0.2 U xanthine oxidase/ml and 0.1 mmol/L EDTA). SOD activity was expressed as unit/milligram of protein with reference to the activity of a standard curve of bovine Cu/Zn SOD under absorbance at 550 nm. (2) CAT activities were determined by estimating a decomposition rate of hydrogen peroxide (H_2_O_2_) in absorbance at 240 nm at 25°C. It was developed by the reaction of 2.0 ml of the supernatant in 26 *μ*ml of 50 mmol/L phosphate buffer (pH 7.0) with 12 *μ*ml of fresh 30 mmol/L H_2_O_2_. CAT activities were expressed as unit/milligram of protein of kidney tissue protein.

To evaluate the scavenging capability of reactive oxygen species (ROS), renal glutathione (GSH) level was quantitated as described previously [41]. Briefly, 4.0 ml of the supernatant was mixed with 20 ml of 0.3 mol/LM Na_2_HPO_4_ and 4.0 ml of 0.04% 5,5-dithiobis-(2-nitrobenzoic acid) in 1% sodium citrate. After 10 min of incubation at room temperature, the concentration of GSH was determined based on the reading intensity of relatively stable yellow under absorbance at 412 nm. It was expressed as milligrams per gram of protein adjusted with known concentrations of GSH under a standard curve.

### 2.10. Determination of Renal Lipid Peroxidation

For the determination, the degree of renal lipid peroxidation, thiobarbituric acid reactive substance (TBARS), and malondialdehyde (MDA) were estimated. (1) TBARS level was measure by OxiSelect™ TBARS assay kit (Koma Biotech Co., Seoul, Korea) according to the manufacturer's instructions. The result was expressed as nanomoles per milligram of protein. (2) MDA level was measured as previously described [42, 43]. Briefly, 1.0 ml of the supernatant was mixed with 2 ml of thiobarbituric acid (0.67%, w/v) under a water bath at 95°C for 45 min. After cooling the mixture to room temperature in an ice bath for 10 min, 900 *μ*l n-butanol was added to extract the MDA-TBA adduct. The upper layer was collected by centrifugation at 16,000 x g for 3 minutes. To dissolve the adduct, 700 *μ*l ddH_2_O was added. The supernatant was measured spectrophotometrically at 532 nm. The result was expressed as nanomoles per milligram of protein.

### 2.11. Western Blotting for Intrarenal Proapoptotic Protein Caspase-9

To evaluate the effect of anthocyanins on renal apoptotic process, we detected one of central effector caspases—caspase-9. The method has been previously described. [44, 45]. First, the kidney samples were homogenized in ice-cold lysis buffer which contains 200 mmol/L NaCl, 10 mmol/L Trisbase, 5 mmol/L MEDTA, 10% glycerin, 1 mmol/L PMSF, 0.1 U/ml aprotinin, and 1.0 *μ*g/ml leupeptin. Then, the homogenized solution was centrifuged at 3000 rpm for 10 min, and the resulted supernatant was centrifuged at 10,000 rpm for 3 min. The final supernatants were collected. Its total protein concentrations were determined using the BCA assay kit (Nanjing Jiancheng Biotechnology Co., Ltd). For Western blotting, the aliquots of kidney protein were heated at 95°C for 5 min in SDS buffer (50 mmol/L Trisbase (pH 6.8), 0.5% glycerol, 0.01% bromophenol blue, and 0.75% SDS) and then separated on 15% SDS–polyacrylamide gels and transferred to a nitrocellulose membrane (Millipore, Bedford, MA). The transferred membrane was blocked with 5% nonfat dry milk in TBST buffer (50 mmol/L Tris (pH 7.5), 150 mmol/L NaCl, and 0.1% Tween) overnight at 4°C. Immunoblot was performed by using a rabbit anti-caspase-9 polyclonal (1 : 1000 dilution) (Santa Cruz Biotechnology, CA). After incubation, the membrane was washed in PBS with 3% nonfat dry milk and 0.05% Tween for 1 h at room temperature and further incubated with the horseradish peroxidase-conjugated anti-rabbit immunoglobulin G for 1 h at room temperature, and the subsequent result was better visualized by enhanced chemiluminescence Western blotting detection reagent (Supersignal, Pierce, Rockford, IL), according to the manufacturer's instructions. The film of blotting bands was quantified densitometrically by computer-designed display camera and image analysis software (Bio-Rad Laboratories, Hercules, CA). To ensure equal protein loading, immunoblotting was performed with an anti-*β*-actin antibody (Sigma, Chicago, IL). To confirm molecular mass, prestained protein markers (Bio-Rad Laboratories, Hercules, CA) were carried out.

### 2.12. Histopathology

For histopathology examination, kidney samples were fixed in a 10% buffered formalin solution and embedded in paraffin, and then the sections (5 *μ*m) were stained with hematoxylin and eosin. To evaluate the degree of tubular injury, the histopathology scoring was carried out by two specialists in pathology who were unaware of experimental groups. The calculation was based on the percentage of tubules in the cortico-medullary junction that displayed certain tubular injury criteria. The tubular injury score was calculated according to the criteria: cellular necrosis, a loss of brush border, and cast formation as well as tubular dilatation (×400 in magnification) in randomly chosen nonoverlapping fields per section for each sample. The tubular injury was semiquantitatively scored as mean ± SD from 0 to 5 grades: 0 = none; 1 = minor, ≤10%; 2 = moderate, 11–25%; 3 = severe, 26–45%; 4 = very severe, 46–75%; and 5 = extensive damage, ≥75% [46], and there were at least 10 high-power fields per slide counted.

### 2.13. Analysis of Experimental Results

All measurement variances were expressed as mean ± standard deviation, and differences in parameter values between the groups were determined for statistical significance by ANOVA. The significance between samples was compared at the *P* < 0.05 level using Duncan's multiple range test in the SPSS 12.0 (SPSS Inc., Chicago, IL, USA) statistical program.

## 3. Results

**Figure 1:**
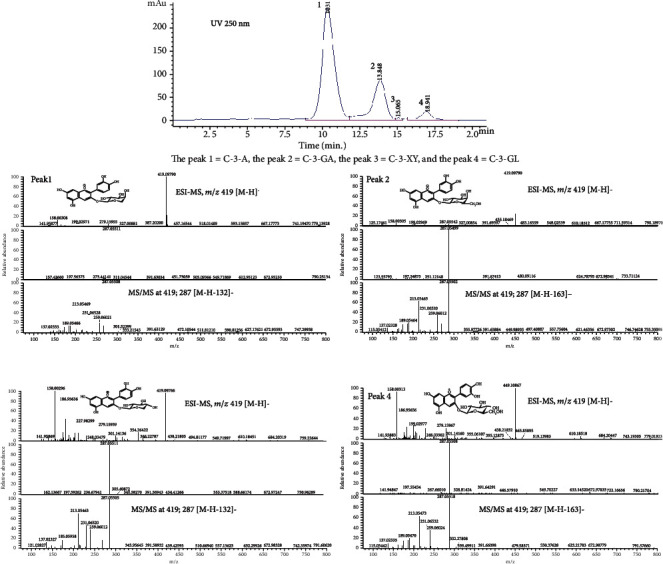
Chromatogram representing the peaks of anthocyanins and the MS/MS analyses of anthocyanin peaks.

The components of *Aronia melanocarpa* anthocyanins were analyzed by UV-Vis, HPLC-DAD, and UPLC-MS methods. Four constituent parts of the anthocyanins were identified as follows: cyanidin-3-arabinoside (68.68%), cyanidin-3-glucoside (5.28%), cyaniding-3-galactoside (25.62%), and cyaniding-3-xyloside (0.42%).  Figure 1 shows HPLC chromatogram of *Aronia melanocarpa* anthocyanins after purified by HP-20 resin at 520 nm.

The experimental process and perioperative period were well tolerated, and no animal died during the two-week experiment. The body weights of mice have no significant change (Table 1 in Supplemental material).

### 3.1. Renal Function Measurements

**Figure 2:**
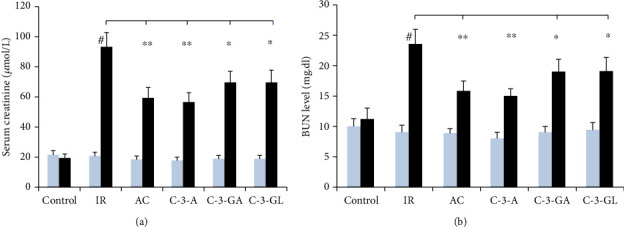
Serum creatinine and blood urea nitrogen levels in the renal IR injury and anthocyanin treatment. The data are the means ± SD. *n* = 13 per group. IR: ischemia/reperfusion; AC: anthocyanins; C-3-A: cyanidin-3-arabinoside; C-3-GA: cyanidin-3-galactoside; C-3-GL: cyanidin-3-glucodise. The comparison between treated mice and IR was indicated by asterisks. ^#^*P* < 0.01 vs. control within group; ^∗^*P* < 0.05 and ^∗∗^*P* < 0.01 vs. respective IR group.

The values of sCr and BUN appear in Figure 2. The renal IR injury resulted in an increase in serum creatinine (sCr) with a peak of 92.9 ± 9.9 *μ*mol/L and blood urea nitrogen (BUN) with a peak of 23.5 ± 2.4 nmol/l compared to the control group. Treatment with anthocyanins significantly reduced the elevated sCr and BUN in comparison with the levels observed in the IR group. Among the anthocyanins, AC and C-3-A exhibited a relatively higher effect than C-3-GA and C-3-GL.

### 3.2. Proinflammatory Cytokines in Serum and Kidney

**Figure 3:**
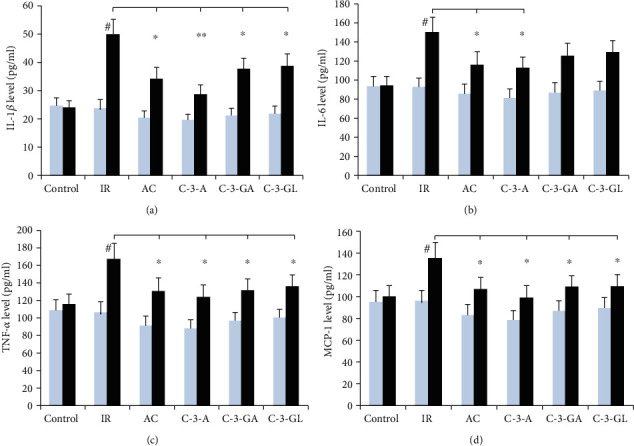
Proinflammatory cytokine's levels in blood in the renal IR injury and anthocyanin treatment. The data are the means ± SD. *n* = 13 per group. (a) IL-1*β*: interleukin-1 beta. (b) IL-6: interleukin-6. (c) TNF-*α*: tumor necrosis factor-*α*. (d) MCP-1: monocyte chemoattractant protein. IR: ischemia/reperfusion; AC: anthocyanins; C-3-A: cyanidin-3-arabinoside; C-3-GA: cyanidin-3-galactoside; C-3-GL: cyanidin-3-glucodise. The comparison between treated mice and IR was indicated by asterisks. ^#^*P* < 0.01 vs. control within group; ^∗^*P* < 0.05 and ^∗∗^*P* < 0.01 vs. respective IR group.

The effects of anthocyanins on proinflammatory cytokine in serum are shown in Figure 3. Anthocyanin treatment had no significant impact in levels of IL-1*β*, IL-6, TNF-*α*, and MCP-1 before the renal IR injury. Compared to the sham-control group, the renal IR group significantly revealed higher levels of these cytokines in serum (IL-1*β*: 49.9 ± 5.2 vs. 28.7 ± 3.2 pg/ml; IL-6: 149.8 ± 15.2 vs. 93.9 ± 9.5 pg/ml; TNF-*α*: 168.6 ± 17.1 vs. 115.9 ± 12.9 pg/ml; and MCP-1 : 135.6 ± 13.3 vs. 99.6 ± 10.1 pg/ml, *P* < 0.01). Nevertheless, anthocyanin treatment reduced the elevated levels of IL-1*β*, TNF-*α*, and MCP-1. Additionally, the elevated IL-6 level was significantly reduced in AC and C-3-A treatments but not in C-3-GA and C-3-GL treatments.

**Figure 4:**
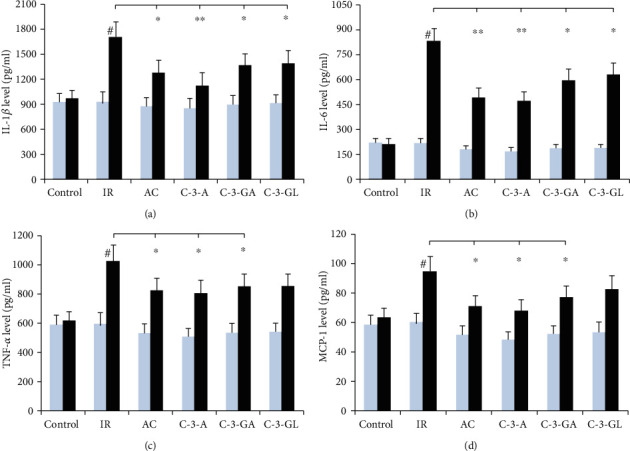
Proinflammatory cytokine's levels in the kidney of the renal IR injury and anthocyanin treatment. The data are the means ± SD. *n* = 13 per group. TNF-*α*: tumor necrosis factor-*α*; IL-1*β*: interleukin-1 beta; IL-6: interleukin-6; MCP-1: monocyte chemoattractant protein; IR: ischemic reperfusion; AC: anthocyanins; C-3-A: cyanidin-3-arabinoside; C-3-GL: cyanidin-3-glucodise; C-3-GA: cyanidin-3-galactoside. ^#^*P* < 0.01 vs. control within group; ^∗^*P* < 0.05 and ^∗∗^*P* < 0.01 vs. respective IR group.

**Figure 5:**
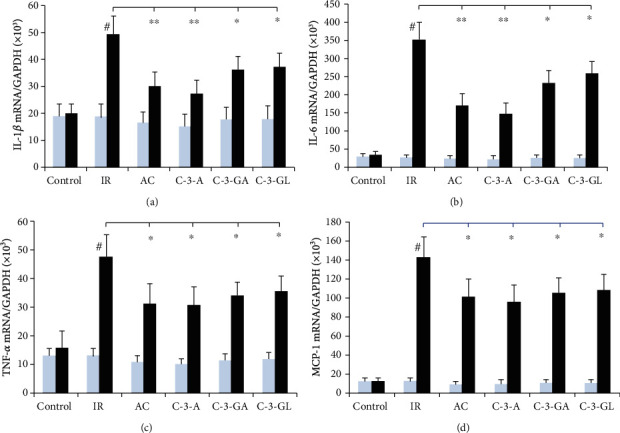
Proinflammatory cytokine's mRNA expression in the renal IR injury and anthocyanin treatment. The data are the means ± SD. *n* = 13 per group. TNF-*α*: tumor necrosis factor-*α*; IL-1*β*: interleukin-1 beta; IL-6: interleukin-6; MCP-1: monocyte chemoattractant protein; IR: ischemic reperfusion; AC: anthocyanins; C-3-A: cyanidin-3-arabinoside; C-3-G: cyanidin-3-glucodise; C-3-GA: cyanidin-3-galactoside. ^#^*P* < 0.01 vs. control within group; ^∗^*P* < 0.05 and ^∗∗^*P* < 0.01 vs. respective IR group.

The effects of anthocyanins on proinflammatory cytokines in the kidney are shown in Figures 4 and 5. There was no significant difference in the mRNA and protein levels of these cytokines in the kidney between the control group and anthocyanin treatment groups before the renal IR injury. The renal IR injury significantly increased the mRNA and protein levels of these cytokines in the kidney, compared to the sham-control group. However, AC, C-3-A, and C-3-GA treatments reduced the elevated mRNA and protein levels of these cytokines in the kidney. Additionally, there was no significant effect on protein levels of TNF-*α* and MCP-1 in the kidney by 6 C-3-GL treatments.

### 3.3. Renal Antioxidant Parameter Levels

**Figure 6:**
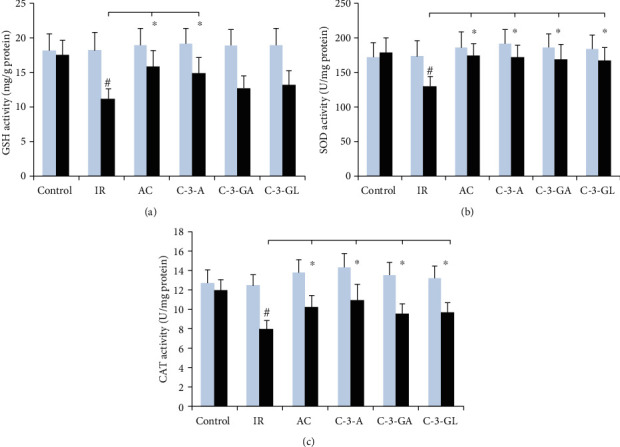
Antioxidant parameter levels in the kidney of the renal IR injury and anthocyanin treatment. The data are the means ± SD. *n* = 13 per group. GSH: glutathione; SOD: superoxide dismutase; CAT: catalase; IR: ischemic reperfusion; AC: anthocyanins; C-3-A: cyanidin-3-arabinoside; C-3-G: cyanidin-3-glucodise; C-3-GA: cyanidin-3-galactoside. ^#^*P* < 0.01 vs. control within group; ^∗^*P* < 0.05 vs. respective IR group.

The results of GSH, SOD, and CAT in the kidney are illustrated in Figure 6. The levels of GSH and activities of SOD and CAT in the renal IR injury were markedly reduced compared to the sham-control group (GSH: 11.1 ± 1.6 vs. 17.4 ± 0.8 mg/g; SOD: 130.8 ± 13.6 vs. 174.1 ± 20.6 U/mg; CAT: 8.0 ± 0.8 vs. 12.5 ± 1.1 U/mg, *P* < 0.01). Anthocyanin treatment has significantly higher levels of SOD and CAT than did the IR injury group. Similarly, the increased level of GHS was seen in AC and C-3-A but not in C-3-GA and C-3-GL treatment groups.

### 3.4. Determination of Renal Lipid Peroxidation

**Figure 7:**
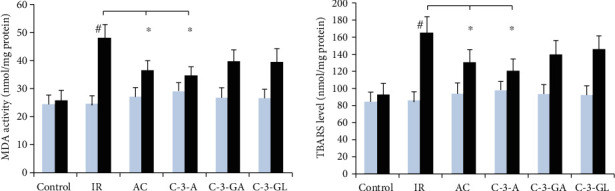
Lipid peroxidation markers in the kidney of the renal IR injury and anthocyanin treatment. The data are the means ± SD. *n* = 13 per group. MDA: malondialdehyde; TBARS: thiobarbituric acid reactive substance; IR: ischemic reperfusion; AC: anthocyanins; C-3-A: cyanidin-3-arabinoside; C-3-G: cyanidin-3-glucodise; C-3-GA: cyanidin-3-galactoside. ^#^*P* < 0.01 vs. control within group; ^∗^*P* < 0.05 vs. respective IR group.

The results of lipid peroxidation byproducts MDA and TBARS in the kidney are illustrated in Figure 7. MDA and TBARS levels in the IR injury group were significantly higher than that in the sham-control group (MDA: 24.8 ± 2.9 vs. 48.3 ± 4.5 nmol/mg; TBARS: 85.2 ± 11.5 vs. 164.9 ± 19.1 nmol/mg, *P* < 0.01). MDA and TBARS levels in AC and C-3-A treatment groups were significantly lower than that in the IR injury group. There was no difference in the levels of MDA and TBARS between C-3-GA or C-3-GL and the IR injury group.

### 3.5. Toll-Like Receptor 4 (TLR4) mRNA Expression

**Figure 8:**
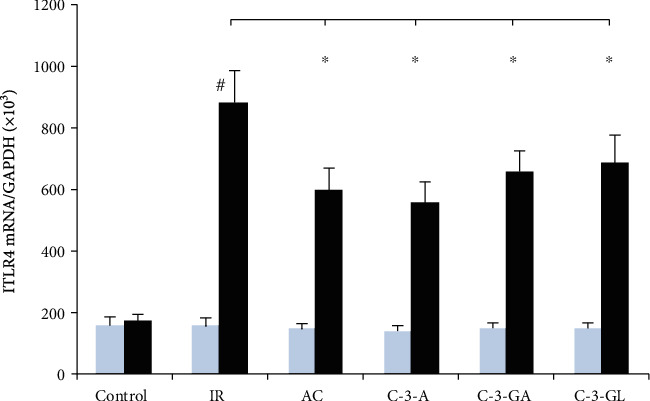
TLR4 mRNA expression in the kidney of the renal IR injury and anthocyanin treatment. TLR4: toll-like receptor 4. The data are the means ± SD. *n* = 13 per group. IR: ischemia/reperfusion; AC: anthocyanins; C-3-A: cyanidin-3-arabinoside; C-3-G: cyanidin-3-glucodise; C-3-GA: cyanidin-3-galactoside. The comparison between treated mice and IR was indicated by asterisks. ^#^*P* < 0.01 vs. control within group; ^∗^*P* < 0.05 vs. respective IR group.

Figure 8 shows the TLR4 mRNA expression in the kidney. The IR injury significantly induced renal TLR4 mRNA expression. Treatments with anthocyanins significantly reduced the upregulation in expression compared to the IR injury group.

### 3.6. Evaluation of Apoptosis Mediator-Caspase-9 Expression

**Figure 9:**
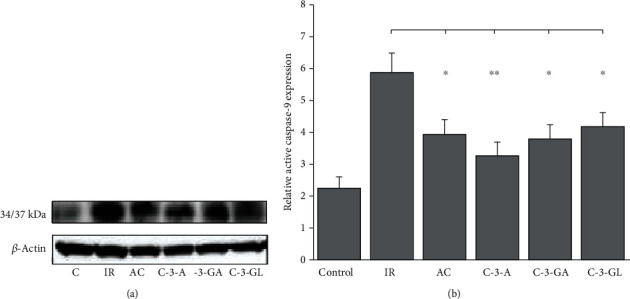
Caspase-9 expression in the kidney of the renal IR injury and anthocyanin treatment. (a) Representative Western blot of intrarenal activated cleaved caspase-9. (b) Densitometric analysis of caspase-9 expression (the means ± SD, *n* = 13 per group). IR: ischemia/reperfusion; AC: anthocyanins; C-3-A: cyanidin-3-arabinoside; C-3-G: cyanidin-3-glucodise; C-3-GA: cyanidin-3-galactoside. The comparison between treated mice and IR was indicated by asterisks. ^#^*P* < 0.01 vs. control; ^∗^*P* < 0.05 and ^∗∗^*P* < 0.01 vs. IR.

Figure 9 shows the Western blotting and densitometric result of the caspase-9 expression in the kidney. Consistent with the levels of TLR4 mRNA in the kidney, the caspase-9 (34/37 kDa) in the IR injury group was significantly higher, by 2.2-fold (*P* < 0.01), than that in the sham-control group. Treatments with anthocyanins markedly reduced the level of caspase-9 compared to the IR injury group.

### 3.7. Histopathological Evaluation

**Figure 10:**
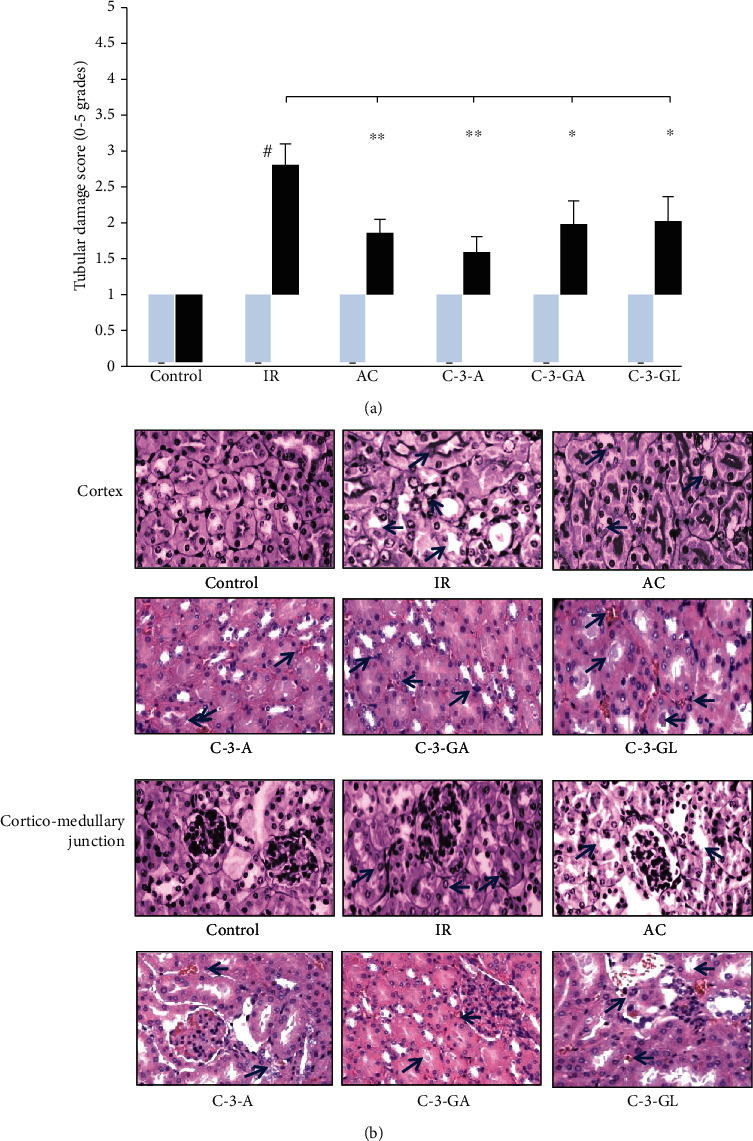
Histopathological evaluation in the renal IR injury and anthocyanin treatment. (a) Quantitative evaluation of renal tubular damage. IR: ischemia/reperfusion; AC: anthocyanins; C-3-A: cyanidin-3-arabinoside; C-3-G: cyanidin-3-glucodise; C-3-GA: cyanidin-3-galactoside. Each group has two scores, one represented before the IR injury (light bars); another represented after the IR injury (black bars). The comparison between treated mice and IR was indicated by asterisks. The data are the means ± SD. *n* = 7 per group. Histopathological scoring of tubular injury was concomitant with histologic analysis for each experimental group. Data are expressed as mean ± SD. ^#^*P* < 0.01 vs. control; ^∗^*P* < 0.05 and ^∗∗^*P* < 0.01 vs. IR group. (b) Representative histologic sections of the kidney including cortex and cortico-medullary junction (H&E at ×400). Sham-control group: normal renal tubules with normal cytoplasm and nucleus and no interstitial edema; IR group: the loss of brush border, tubular lumen dilation, vacuolation, and degeneration, shown as blue arrow; AC, C-3-A, C-3-GA, and C-3-GL groups: mild tubular degeneration and edema and trace tubular degeneration, indicated as blue arrow.

Figure 10 shows the effect of anthocyanins on the histopathology and tubular structure damage. Compared to the sham-control group, the kidney of the IR injury group revealed the loss of brush border and tubular lumen dilation as well as necrosis. The morphological changes in anthocyanin treatment were shown to a milder degree. The tubular damage score shows that the lesion in the IR injury group was alleviated with anthocyanin treatment.

## 4. Discussion

The fundamental purpose of the current study was to explore the preventive effect of specific anthocyanins on kidney ischemia-reperfusion injury. Out studies show that the oral pretreatment with anthocyanins alleviated renal dysfunctions in the acute injury model in vivo. The initial finding is supported by two observations. First, pretreatment of IR mice with anthocyanins reduced the increased levels of serum creatinine and blood urea nitrogen compared to the nontreated. Second, anthocyanins attenuated the pathological lesion characterized by tubular cell swelling and dilatation and necrosis as well as glomerular atrophy. Of the different anthocyanins, cyanidin-3-arabinoside was the most effective and cyanidin-3-glucoside was the slightest.

The second purpose of the study was to investigate their potential mechanisms in the effect against renal IR injury. Renal IR injury is characterized by deficient oxygen supply and subsequent restoration of blood flow and irreversible damages to microvascular and parenchymal cells.

Both renal mRNA quantitative analysis and activity measurement revealed a considerable elevation in several inflammatory cytokines IL-1*β*, IL-6, TNF-ɑ, and MCP-1 after renal IR injury compared to the control, whereas anthocyanins contained the elevation. Accumulative evidences showed inflammatory elevation could be one of the important etiological mechanisms of renal IR injury [47–49]. IL-1*β* is a critical inflammatory mediator, and its activation in turn stimulates the release of additional inflammatory cytokines IL-6 and TNF-ɑ, causing local inflammation and massive apoptosis of nephrocytes [50] as well extensive tubular damage [48, 49]. MCP-1 is a dominant inflammatory component in recruiting monocytes, macrophages, and T lymphocytes to kidney injury [51]. Thus, anthocyanin-mediated protection against renal IR injury in vivo is related to global and renal inflammatory cascade.

Toll-like receptor 4 (TLR4) plays a critical role in renal IR injury because it activates the inflammatory reaction through NF-*κ*B activation [52, 53]. NF-*κ*B is a transcription factor that induces the production of proinflammatory factors, such as IL-1*β* and TNF-*α* and subsequent kidney damage [54, 55]. Also, TLR4 signaling activation is positively linked to iNOS, resulting in the excessive peroxynitrite formation in renal vascular smooth muscle cells and tubular cells [56]. The finding of downregulation of TLR4 mRNA expression during renal IR injury by anthocyanin suggests that anthocyanins confer renal protection from IR injury through inhibition of iNOS expression and inflammatory reaction.

ROS-medicated oxidative stress is implicated in the complex process of renal IR injury [57] including attacking the lipid to initiate free radical chain reactions and lipid peroxidation in the kidney [58]. Antioxidant enzymes GSH, SOD, and CAT play important role as endogenous scavenger in reducing oxidative stress [59, 60]. MDA and TBARS are reported as two of indicators of lipid oxidation caused by oxidative stress [61, 62]. Oxidative stress and lipid peroxidation analysis revealed a significant decrease in levels of GSH, SOD, and CAT and an elevation in MDA and TBARS levels by IR whereas anthocyanins prevented most alterations. These results suggest that the preventive effect of anthocyanins is ascribed to increase the activity of antioxidant enzymes and suppress lipid peroxidation in renal IR injury. Our findings are consistent with previous studies indicating antioxidant capacity and attenuation of lipid peroxidation with fruit extraction from black chokeberry [63, 64].

In renal IR injury, fragmented mitochondria increase significantly in renal tubule cells, which compromises energy metabolism and aggravates production of reactive oxygen species (ROS) and apoptosis. Mitophagy is a selective degradation of mitochondria via autophagy against renal tubular cell death [65]. Our finding that anthocyanins protected renal tubular score suggests anthocyanins may promote endogenous mitophagy activity to enable tubular cell survival during IR injury.

Previous studies showed that the cascapse-9 is an important marker for intrinsic apoptosis during the induction of renal IR damage [66–68]. An increase in cleaved cascapse-9 expression was suppressed by anthocyanin treatment compared with untreated group, suggesting nephroprotective effect of anthocyanins in IR injury is related to blockage of intrinsic apoptosis cascade process. However, further study should be required to investigate different effects of anthocyanins on distal tubules and proximal tubule associated with caspase-medicated pathway and DNA damage [69].

In renal IR injury, a series of pathological development and progression not only involved with local inflammatory process but also hemodynamic unstabilization [47, 70, 71]. The 2-week oral administration indicates that a certain time is required for anthocyanin to be effective in the prevention of renal IR injury. Furthermore, it is possible that anthocyanins may modify the balance between immune effector cells and suppressor cells in circulation; thus, it decreases susceptibility to renal IR injury and benefits repair processes to a certain extent.

Overall, anthocyanins ameliorate renal IR injury in vivo through anti-inflammation and reduction in oxidative stress and lipid peroxidation. This is the first prospective observation to our knowledge that anthocyanins from black chokeberry have the potential to prevent acute renal IR injury in vivo. Among these anthocyanins, the nephroprotective effect was different with the relatively higher with cyanidin-3-arabinoside and relatively lower with cyanidin-3-glucoside. Its variance in efficiency may be reflected by their molecular structures and different secondary metabolic pathways. Moreover, further investigation is required to determine anthocyanins' metabolism and degradation associated with the prophylactic role in renal IR injury incidence and clinical outcomes.

## 5. Conclusions

In conclusion, this data provided a further referenced role of anthocyanin exposure in nephroprotective effect on acute IR injury in mice. The findings support unique properties of anthocyanins against inflammation, oxidative stress, and lipid peroxidation occurred in renal injury. The current study demonstrated feasibility with anthocyanins for prevention in acute renal injury.

## Figures and Tables

**Table 1:** The primer sequence for RT-qPCR.

The primer sequences	Forward	Reverse
IL-1*β*	5′-GCACACCCACCCTGCAG-3′	5′-AACCGCTTTTCCATCTTCTTCTT-3′
IL-6	5′-GATGCTACCAAACTGGATATAATC-3′	5′-GGTCCTTAGCCACTCCTTCTGTG-3′
TNF-ɑ	5′-CCATTCCTGAGTTCTGCAAAG-3′	5′-GCAAATATAAATAGAGGGGGGC-3′
MCP-1	5′-GAGCATCCACGTGTTGGCT-3′	5′-TGGTGAATGAGTAGCAGCAGGT-3′
TLR4	5′-CGC TTT CAC CTC TGC CTT CAC-3′	5′-TTG CCG TTT CTT GTT CTT CTT C-3′

## Data Availability

The data used to support the findings of this study are included within the article.

## Supplementary Materials

Supplementary Materials Supplementary Material Figure 1: chemical structure of *Aronia melanocarpa* anthocyanins. Supplementary Material Table 1: body weight in the renal IR injury and anthocyanin treatment.
