# Supplementary material for: The Protective Effect of Anthocyanins Extracted from Aronia Melanocarpa Berry in Renal Ischemia-Reperfusion Injury in Mice
Source: Mediators Inflamm. 2021 Jan 22;2021:7372893. doi: 10.1155/2021/7372893 (PMC7846408; doi:10.1155/2021/7372893)
Supplement: Supplementary Materials — Supplementary Material Figure 1: chemical structure of Aronia melanocarpa anthocyanins. Supplementary Material Table 1: body weight in the renal IR injury and anthocyanin treatment. [file 7372893.f1.docx]

**Supplementary Material for ID 7372893**

Supplementary Material **Figure 1. Chemical structure of *Aronia melanocarpa* anthocyanins**


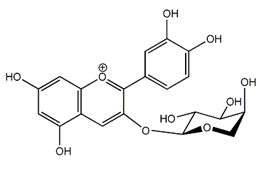

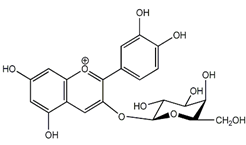

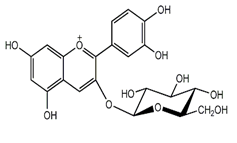

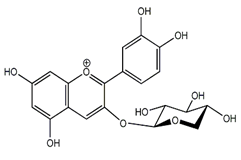


**A**

**B**

**C**

**D**

Figure 1_Legend: A, cyanidin-3-arabinoside; B, cyanidin-3-galactoside; C, cyanidin-3-glucoside; and D, cyanidin-3-xyloside.

Supplementary Material **Table 1. Body weight in the renal IR injury and anthocyanins treatment**

| Time/ Group | Control (n=13) | IR (n=13) | Cyanidin/IR treated | | | |
| --- | --- | --- | --- | --- | --- | --- |
|  |  |  | AC (n=13) | C-3-A (n=13) | C-3-GA (n=13) | C-3-GL (n=13) |
| Before treatment | 20.2 ± 1.0 | 20.8 ± 1.1 | 20.5 ± 0.9 | 20.4 ± 1.1 | 20.2 ± 0.9 | 21.0 ± 1.1 |
| After treatment | 20.1 ± 0.9 | 20.7 ± 0.8 | 20.8 ± 1.2 | 21.0 ± 1.0 | 20.6 ± 0.8 | 20.5 ± 0.7 |
| After IR | 18.9 ± 1.0 | 18.7 ± 0.9 | 18.6 ± 0.8 | 18.7 ± 1.2 | 18.6 ± 1.1 | 18.5 ± 1.1 |
|  | | | | | | |

Table 1_Legend: The data are the means +-SD. n=13 per group. After IR, 24 hours after ischemia/reperfusion model. AC: Anthocyanins, C-3-A: cyanidin-3-arabinoside, C-3-GA: cyanidin-3-galactoside, C-3-GL: cyanidin-3-glucodise.
